# Greater Breast Support Is Associated With Reduced Oxygen Consumption and Greater Running Economy During a Treadmill Running Task

**DOI:** 10.3389/fspor.2022.902276

**Published:** 2022-06-14

**Authors:** Hailey B. Fong, Douglas W. Powell

**Affiliations:** Breast Biomechanics Research Center, College of Health Sciences, University of Memphis, Memphis, TN, United States

**Keywords:** running, treadmill, bioenergetics, VO_2_, oxygen consumption, running economy, breast, sports bra

## Abstract

**Introduction:**

Breast pain is a major barrier to running for women. While breast support through the use of sports bras reduces breast-related discomfort, the effect of breast support on running performance is less understood. Therefore, the purpose of the current study was to evaluate the effect of greater breast support on oxygen consumption and running economy during a treadmill running task.

**Methods:**

Fifteen female recreational runners performed a 10-min treadmill running task at their preferred running speed in each of two sports bra conditions: low support and high support. Participants ran on an instrumented treadmill (1,200 Hz, Bertec) while indirect calorimetry was performed using a metabolic measurement system (100 Hz, TrueOne, ParvoMedics). Average VO_2_ (absolute and relative) from the third to 10th minutes was used to evaluate oxygen consumption. Running economy was calculated as the distance traveled per liter of oxygen consumed. Paired samples *t*-tests were used to compare mean oxygen consumption and running economy values between breast support conditions. Correlation analysis was performed to evaluate the relationship between breast size and change in running performance.

**Results:**

Greater breast support was associated with reductions in absolute (*p* < 0.001) and relative oxygen consumption (*p* < 0.001; LOW: 30.9 ± 7.1 ml/kg/min; HIGH: 28.7 ± 6.7 ml/kg/min). Greater breast support was associated with increases in running economy (*p* < 0.001; LOW: 88.6 ± 29.1 m/L O_2_; HIGH: 95.2 ± 31.1 m/L O_2_). No changes in temporospatial characteristics of running were observed including cadence (*p* = 0.149), step length (*p* = 0.300) or ground contact time (*p* = 0.151). Strong positive linear correlations were observed between the change in running performance metrics and breast size (Oxygen Consumption: *p* < 0.001, *r* = 0.770; Relative Oxygen Consumption: *p* < 0.001, *r* = 0769; Running Economy: *p* < 0.001, *r* = 0.807).

**Conclusions:**

Greater breast support was associated with reduced oxygen consumption and increased running economy. These findings demonstrate that greater breast support is not only associated with improved comfort but also improved running performance.

## Introduction

Running is a popular form of physical activity that has been shown to benefit cardiovascular and musculoskeletal health (Williams et al., 1984; Piacentini et al., 2013; Lavie et al., 2015; Kozlovskaia et al., 2019). For many women, however, breast pain is a significant barrier to exercise including running-based activities (Scurr et al., 2011a; Risius et al., 2017; Brisbine et al., 2020; McGhee and Steele, 2020b). It is reported that up to 72% of females experience breast pain during exercise-related activities (Gehlsen and Albohm, 1980; Bowles et al., 2008; Scurr et al., 2010). Several factors are suggested to underlie exercise-induced breast pain including large breast displacements as well as high breast velocities and accelerations (McGhee and Steele, 2020a). Moreover, these breast displacement magnitudes are influenced by breast size with larger breasts experiencing greater breast displacement magnitudes compared to smaller breasts (McGhee and Steele, 2010a; McGhee et al., 2013). For example, females with a D-cup breast size can experience vertical breast displacements as high as 20 cm when running without breast support (McGhee et al., 2007; Scurr et al., 2011a). However, when external breast support was provided in the form of a sports bra, vertical breast displacements decreased (Scurr et al., 2011a,b; Risius et al., 2017), attenuating perceived breast pain and improving breast comfort (Scurr et al., 2011b; Milligan et al., 2015; Risius et al., 2017; McGhee and Steele, 2020b).

Breast excursion velocity is another factor that contributes to breast discomfort and breast pain. During running, the breasts experience high magnitudes of excursion velocity in the downward direction (McGhee et al., 2007; Scurr et al., 2009; McGhee and Steele, 2020a) which are purported to be the primary cause of breast pain and discomfort during running. These high vertical breast excursion velocities are created by the difference in timing between the vertical motion of the trunk and breasts. As the trunk decelerates following initial contact, the breasts continue their downward progression at a greater rate than the trunk resulting in the breasts forcefully contacting the anterior trunk wall (McGhee et al., 2007; Scurr et al., 2009; McGhee and Steele, 2020a). A study by McGhee et al. (2007) revealed that females with a C-cup breast size or greater experienced peak breast velocities of 80 cm/s in the upward direction and 100 cm/s in the downward direction during treadmill running. However, when breast velocities were reduced by performing deep water running, running-related breast pain was reduced (McGhee et al., 2007). Due to the greater breast displacement magnitudes associated with larger breast sizes, breast excursion velocities are also proposed to be greater in women with larger breasts (McGhee and Steele, 2010a; McGhee et al., 2013). Therefore, interventions reducing breast displacements and the resultant breast excursion velocities should disproportionately reduce breast pain in individuals with larger breasts.

Greater breast support also alters trunk and pelvis biomechanics during treadmill running. During running, transverse plane rotations of the trunk and pelvis act to balance rotational momentum about the body's center of mass while pelvis rotation is an important contributor to step length and subsequently cadence (Preece et al., 2016). However, Milligan et al. (2015) reported that greater breast support was associated with increased transverse plane trunk and pelvis excursions during treadmill running. Further, greater breast support was associated with increased vertical oscillations of the trunk and pelvis (Risius et al., 2017). These support-related changes in running biomechanics are indicative of a less constrained neuromuscular system, believed to be the result of reduced breast excursions and excursion velocities associated with greater breast support (Milligan et al., 2015; Risius et al., 2015, 2017). A potentially important change in running biomechanics with increasing levels of breast support relates to changes in the vertical oscillations of the trunk and pelvis which may be indicative of altered metabolic cost of running.

The influence of breast support on the bioenergetics of running have not been well established. Only a single study has directly investigated the effect of increasing levels of breast support on the metabolic cost of treadmill running (Risius et al., 2017) and reported no significant changes in variables associated with running bioenergetics including heart rate, oxygen consumption, running economy, minute ventilation or breathing frequency. However, this study had a relatively small sample size of 10 participants with an unreported level of running experience. The effect of breast support on metabolic cost and running economy in female recreational runners remains unknown. Therefore, the purpose of this study is to directly investigate the effect of increasing levels of breast support on oxygen consumption and running economy during a steady-state treadmill running task. It was hypothesized that greater breast support would be associated with reduced oxygen consumption and increased running economy. Further, it was hypothesized that changes in oxygen consumption and running economy would be influenced by breast size.

## Materials and Methods

### Participants

An *a priori* power analysis (G^*^Power 3.1.5) was conducted based on preliminary data of oxygen cost during running in the high- compared to low-support sports bra. Using an effect size of 0.5, an alpha level of 0.05 and power (1-β) of 0.80, a sample size of 15 participants was determined to provide sufficient statistical power for this study. A total of 15 female recreational runners were recruited to participate in the current study. Participants were included if they were: (1) 18–30 years of age, (2) recreational runners with a running mileage >12 miles per week, (3) had a self-reported bra size of B-cup to DD-cup, (4) had no history of prior breast surgeries (augmentation or reduction), (5) had no recent history of lower extremity injury that would negatively affect their running performance (6 months) and (6) were free of injury at the time of testing. All participants had a multi-year history of endurance running with similar or greater running volume than the listed inclusion criteria. The experimental protocol (PRO-FY2020-431) was approved by the University of Memphis Institutional Review Board and all participants provided written informed consent prior to data collection.

### Experimental Protocol

**Figure 1:**
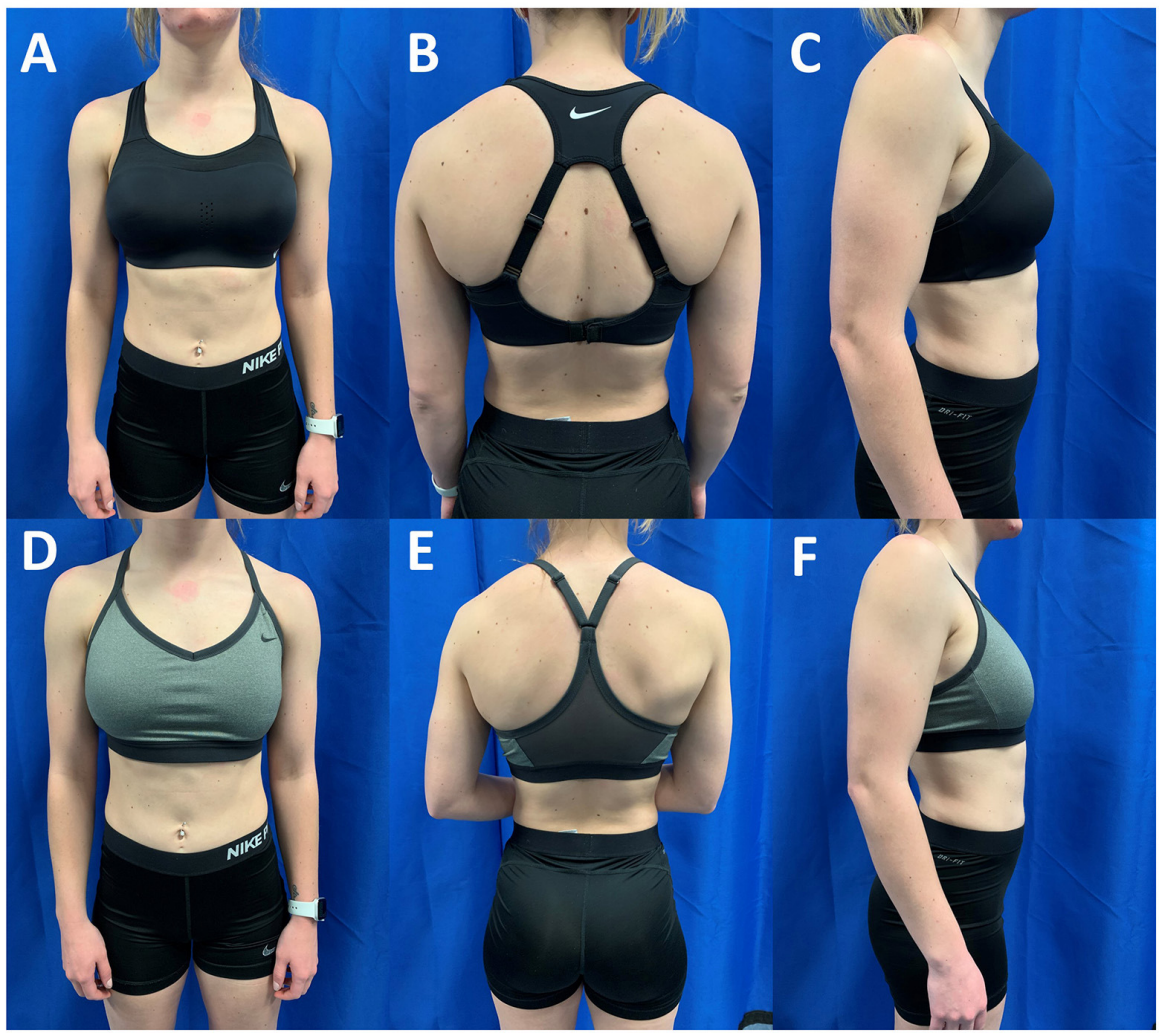
Anterior, posterior and lateral views of a participant with D-Cup sized breasts in the high support Nike Alpha **(A–C)** and low support Nike Indy **(D–F)**. The athlete was classified as a D-Cup based on the difference between her bust and underbust circumferences (Bust: 84 cm; Underbust: 73.5 cm; Difference: 10.5 cm). The high support sports bra is designed to lift and compress the breast tissue while the low support sports bra is not designed with these features.

Age and anthropometric measurements including height (m), mass (kg), and bust and underbust circumferences (cm) and were acquired prior to the running protocol. Each participant's breast size was determined based on the difference between their bust and underbust circumferences (McGhee and Steele, 2010b). One cup size was defined as a one inch (2.54 cm) difference between the bust and underbust circumferences (McGhee and Steele, 2010b). For example, a B-cup was defined as a 2-inch (5.08 cm) bust-underbust difference while a DD-cup was defined as a 5-inch (12.7 cm) bust-underbust difference. Bust and underbust circumferences were measured using a standard retractable measuring tape (ERT-963, Elite Medical Instruments, Orange County, California, USA) while the participant was wearing the low support sports bra provided by researchers. Participants were then provided with the designated size sports bras based on the manufacturer's suggested sizing. Participants verbally confirmed the fit of the sports bra as well as their comfort in the sports bra size. The LOW conditions required the participant to wear a sports bra that is described by the manufacturer as having “light” support for low-impact workouts. The low support sports bras offered the breasts limited support. The low support sports bra was the Nike Dri-FIT Indy (Nike Inc., Beaverton, OR, USA). The sports bra included padding inserts; however, the participants were asked to remove the padding inserts from the sports bra prior to testing. The fabric of the sports bra includes a body and lining made of 88 percent recycled polyester and 12 percent spandex, center back mesh and bottom hem made of 81 percent nylon and 19 percent spandex, elastic made 84–85 percent nylon and 15–16 percent spandex, interlining made of 80 percent polyester and 20 percent spandex, pad top fabric and pad back fabric made of 100 percent polyester, and pad made of 100 percent polyurethane. The HIGH condition required the participant to wear a sports bra that is described by the manufacturer has having their “highest” level of support with a compressive feel for minimal bounce. The high support sports bra was the Nike Dri-FIT Alpha (Nike Inc., Beaverton, OR, USA). The sports bra included both adjustable straps and padding. The fabric of the sports bra includes a body and back lining insets made of 79 percent nylon and 21 percent spandex, mesh and mesh lining made of 81 percent nylon and 19 percent spandex, pad made of 100-polyurethane, and pad back fabric made of 100 percent polyester. Figure 1 demonstrates the differences in support offered by the Nike Indy and Alpha sports bras for a female participant with a breast size of 15.0 cm. Sports bra sizing was completed for both sports bras prior to the beginning of the dynamic testing protocol. The order of presentation of sports bra conditions was randomized between the HIGH and LOW support sports bras.

Prior to dynamic testing, all participants performed a 10-min warm up that consisted of stationary cycling, stretching and light running. Each participant's preferred running speed was then measured over a 20 m runway using an electronic timer and two photocells (63501 IR, Lafayette Instruments Inc., IN, USA). The participant was instructed to run at a pace they would feel comfortable for a “normal” 30-min training run while their running speed was measured over a 3 m distance in the middle of the 20 m runway. The participant's running velocity was used for both experimental conditions.

**Figure 2:**
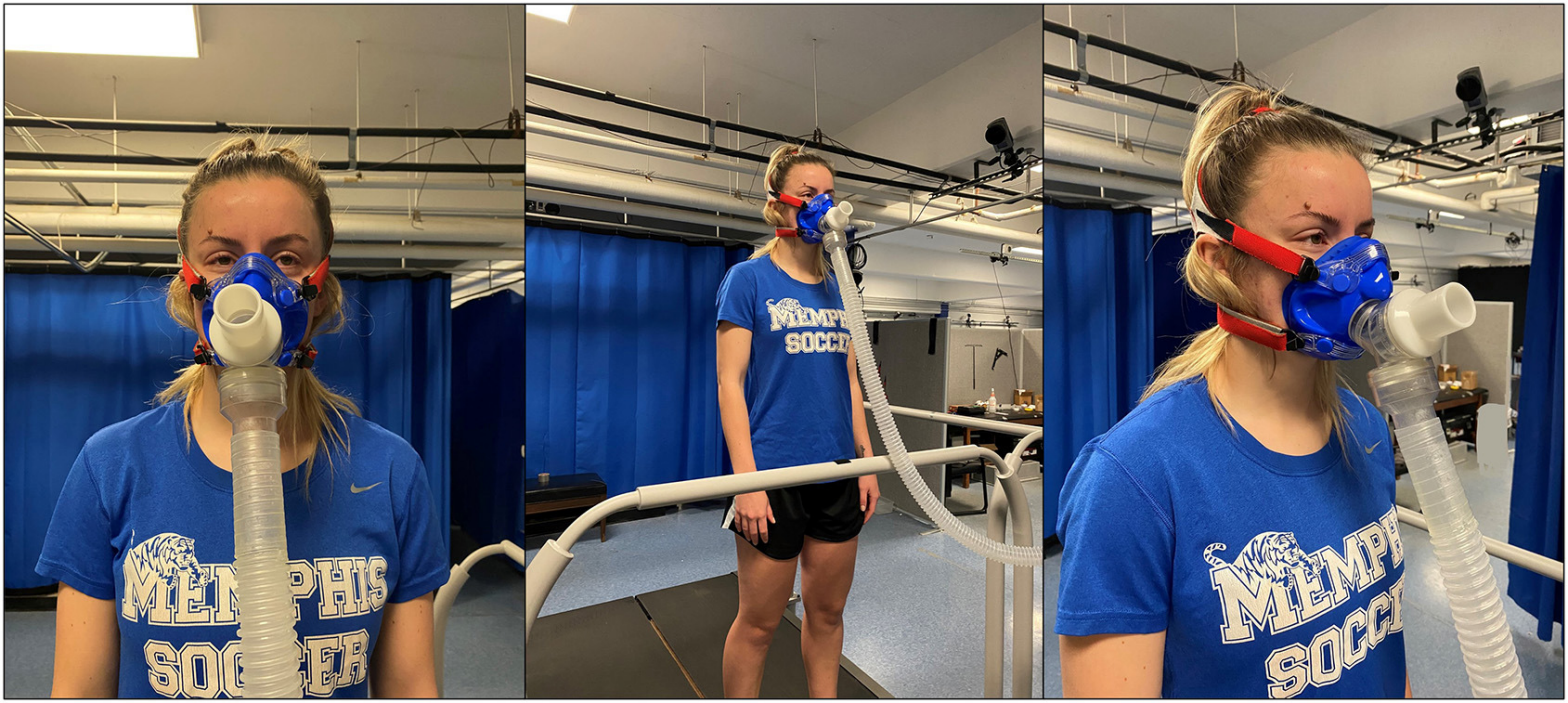
Anterior and anterolateral views of the ParvoMedics TrueOne metabolic measurement system. Expired gases were collected from the participant via a face mask and plastic hose. A proper seal around the mouth and nose was confirmed by research staff prior to testing.

Following completion of the warmup and determining the participant's preferred running speed, participants were fitted to the metabolic measurement system (TrueOne, ParvoMedics, Salt Lake City, Utah, USA). Each participant was placed in a mask that covered their mouth and nose (Hans Rudolph) which was connected to the metabolic measurement system via a plastic tube (Figure 2). The mask was held in place by a series of plastic straps that wrapped around the posterior and superior aspects of the participant's head and were closed via Velcro closures. Prior to initiating dynamic testing, proper fit of the mask on the participant's face was checked by research staff. Proper mask fit was characterized by no air escaping around the sides of the mask when the participant expired air forcefully.

Once the participant had been fitted for the metabolic measurement system, they completed a 10-min treadmill running trial in each of two randomized breast support conditions: LOW and HIGH. During the running trial, oxygen consumption was measured by the metabolic measurement system while temporospatial characteristics were determined from ground reaction force (GRF) data collected using an instrumented treadmill (1200 Hz, Bertec, Split Belt, Columbus, Ohio, USA). The two 10-min running trials were separated by a 10-min period of rest in which the participant was removed from the metabolic measurement system. Following the completion of the 10-min rest period, the experimental protocol was repeated in the other breast support condition.

### Data Analysis

Oxygen consumption (in L/min) for each participant in each condition was represented by the average oxygen consumption between the third and 10th minutes. Oxygen consumption from the first 2 min of each treadmill running trial was discarded as the participant was not considered to be in steady state over this period (Whipp et al., 1982). Relative oxygen consumption for each participant in each condition was calculated as the average oxygen consumption in mL divided by the participant's body mass in kg (ml O_2_/kg/min). Running economy (in m/L O_2_) was calculated as the participant's (treadmill) running velocity (m/s) multiplied by 60 then divided by their oxygen consumption (L/min).

Each participant's cadence, step length and ground contact time were determined from GRF data. GRF data were filtered using a fourth-order, zero-lag Butterworth filter with a 40 Hz cutoff frequency. Initial contact (IC) was defined as the instant in which the vertical GRF signal exceeded a threshold of 50 N for a period >33 ms. Toe off (TO) was defined as the instant at which the vertical GRF decreased below a threshold of 50 N for a period >33 ms. Cadence (in steps/min) was calculated as the total number of ICs (right and left) completed during the recording period divided by the duration of the recording period in minutes (7 min). Step length (in m) was calculated as the time (in seconds) between subsequent ICs (right then left, etc.) multiplied by the treadmill running speed (m/s). Ground contact time (GCT) was calculated as the time (in ms) between the events of IC and TO for the left and right limbs.

### Statistical Analysis

Prior to statistical testing, normality of data for each dependent variable were assessed using a Shapiro-Wilks test. In the presence of a significant Shapiro-Wilks test, non-parametric tests of differences and relationship were used, otherwise, parametric testing was implemented. Parametric tests of differences and relationship included paired samples *t*-tests and Pearson-Product Correlation Coefficients. Non-parametric tests of difference and relationship included Wilcoxon Matched-Pairs Signed Rank test and Spearman Rank Correlations.

Tests of differences were used to compare mean (or median) values for each dependent variable in the HIGH compared to LOW conditions including oxygen consumption, running economy and temporospatial characteristics. Cohen's *d* estimates of effect size were used to evaluate the effect of breast support on oxygen consumption, running economy and temporospatial characteristics (Cohen, 1988). Effect sizes were interpreted as follows: trivial, *d* < 0.02, small, 0.2 < *d* < 0.5, moderate; 0.5 < *d* < 0.8; large, 0.8 < *d*.

The relationships between breast size and breast support-related changes in performance were quantified using Pearson Product correlation coefficients (*r*) or Spearman Rank correlation coefficients (ρ). Coefficients of determination (*r*^2^) were used to evaluate the proportion of variation in performance explained by greater breast support. Statistical analyses were conducted using Prism 9.0 (GraphPad Software, San Diego, CA). Significance was set at *p* < 0.05.

## Results

### Participants

Table 1 presents participant information including age, height, mass, breast size and preferred running speed for each participant. Participants had an average age of 22.0 (±2.3) years, average height of 1.65 (±4.4) m, average mass of 68.1 (±9.7) kg, average breast size of 11.7 (±2.4) cm and an average running speed of 2.9 (±0.4) m/s.

**Table 1:** Individual participant anthropometric data including age, height, mass, breast size and cup size.

**Subject**	**Age**	**Height**	**Mass**	**Breast**	**Cup**	**Speed**
	**(years)**	**(m)**	**(kg)**	**size (cm)**	**size**	**(m/s)**
S1	21	1.67	77.4	15.0	DD	2.6
S2	21	1.69	81.8	12.0	D	2.5
S3	20	1.66	74	9.5	C	2.9
S4	20	1.60	82.4	12.0	D	2.5
S5	27	1.66	68.3	12.5	D	2.7
S6	22	1.62	54.2	13.5	DD	2.5
S7	21	1.70	60.5	14.5	DD	3.5
S8	21	1.64	53.2	10.7	C	3.1
S9	20	1.63	65	12.0	D	3.3
S10	20	1.61	65.5	12.0	D	3.2
S11	24	1.78	76.1	13.5	DD	3.2
S12	24	1.65	77.3	14.0	DD	3.4
S13	26	1.67	57.5	9.0	C	2.8
S14	23	1.63	61.5	7.1	B	3.3
S15	20	1.66	66.3	8.0	C	2.1
Mean	22.0	1.66	68.1	11.7		2.9
SD	2.3	0.04	9.7	2.4		0.4

*Breast size was calculated as the difference between bust and underbust circumferences. Each participant's preferred running speed is also presented*.

### Oxygen Consumption

**Figure 3:**
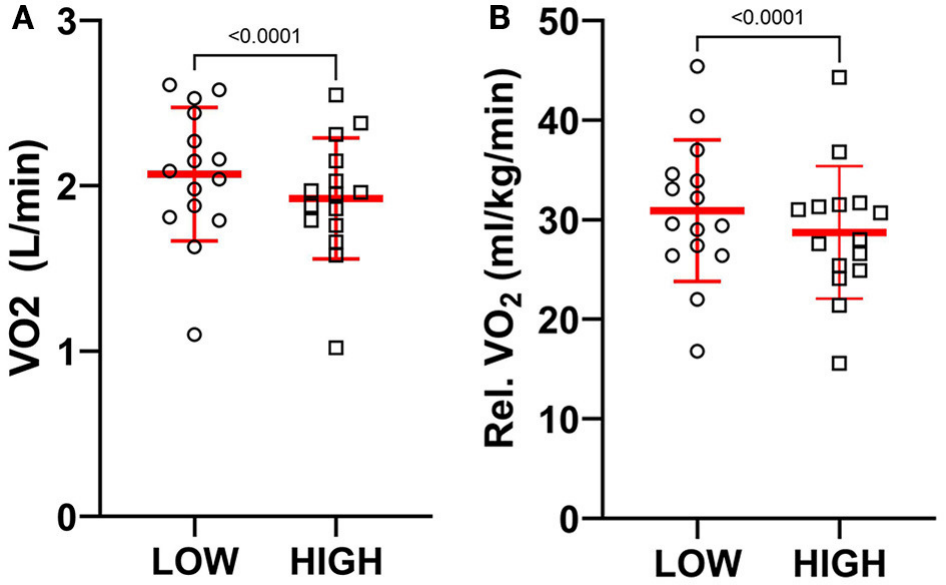
A comparison of individual and mean **(A)** absolute and **(B)** relative oxygen consumption in the LOW and HIGH support conditions during the treadmill running task. A significant reduction in oxygen consumption was observed in the HIGH compared to LOW support conditions.

Table 2 presents oxygen consumption data for each participant during the treadmill running task. The HIGH compared to LOW support condition was associated with reductions in absolute oxygen consumption measured as the average volume of Oxygen consumed (Figure 3A, *p* < 0.001, *t* = 5.79, *d* = 0.39). When normalized to body mass (ml/kg/min), relative oxygen consumption was also lower in the HIGH compared to LOW support condition (Figure 3B, *p* < 0.001, *t* = 5.83, *d* = 0.32).

**Table 2:** Absolute and relative oxygen consumption values as well as relative change in oxygen consumption in the HIGH compared to LOW support conditions are presented.

**Subject**	**Breast**	**LOW**	**HIGH**	**%**	**LOW**	**HIGH**	**%**
	**Size**	**(VO_**2**_**	**(L VO_**2**_**	**Change**			**Change**
	**(cm)**	**L/min)**	**(/min)**				
S1	15.0	2.27	1.97	13.4	29.4	25.4	13.4
S2	12.0	2.16	2.03	6.0	26.4	24.9	6.0
S3	9.5	1.63	1.58	2.6	22.0	21.4	2.6
S4	12.0	2.44	2.31	5.3	29.6	28.0	5.3
S5	21.5	2.53	2.15	15.0	37.0	31.5	15.0
S6	13.5	1.79	1.66	7.2	33.1	30.7	7.2
S7	14.5	2.09	1.88	10.2	34.6	31.0	10.2
S8	10.7	2.15	1.96	8.8	40.4	36.8	8.8
S9	12.0	1.88	1.79	4.7	29.0	27.6	4.7
S10	12.0	1.10	1.02	7.2	16.8	15.6	7.2
S11	13.5	2.58	2.38	7.7	33.9	31.3	7.7
S12	14.0	2.04	1.86	8.7	26.4	24.1	8.7
S13	9.0	2.61	2.55	2.3	45.4	44.3	2.3
S14	7.1	1.98	1.95	1.7	32.2	31.7	1.7
S15	8.0	1.81	1.76	2.7	27.4	26.6	2.7
Mean	12.3	2.07	1.92^*^	6.9	30.9	28.7^*^	6.9
SD	3.5	0.40	0.36	4.0	7.1	6.7	4.0

*The HIGH compared to LOW support sports bra was associated with reduced absolute (p < 0.001, t = 5.79) and relative oxygen consumption (p < 0.001, t = 5.83)*.

*Note:*

* *- denotes a significant difference compared to the LOW condition*.

**Figure 4:**
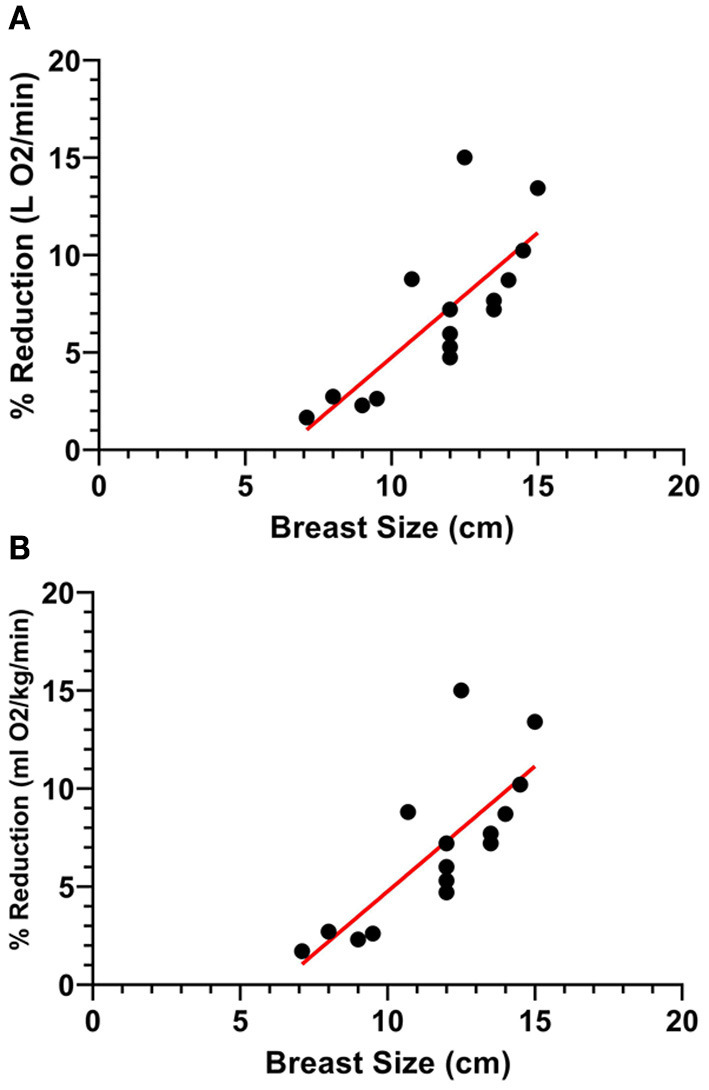
A strong, significant positive relationship was observed between participant breast size (cm) and breast support-induced reductions in **(A)** absolute and **(B)** relative oxygen consumption during the treadmill running task.

Figures 4A,B present the relationship between breast size (in cm) and reductions in (A) absolute and (B) relative oxygen consumption during the treadmill running task. A significant positive correlation was observed between breast size and reductions in absolute oxygen consumption in the HIGH compared to LOW support condition (*p* < 0.001, *r* = 0.770, *r*^2^ = 0.592). A similar significant positive correlation was observed between breast size and reductions in relative oxygen consumption in the HIGH compared to LOW support condition (*p* < 0.001, *r* = 0.769, *r*^2^ = 0.591).

### Running Economy

**Figure 5:**
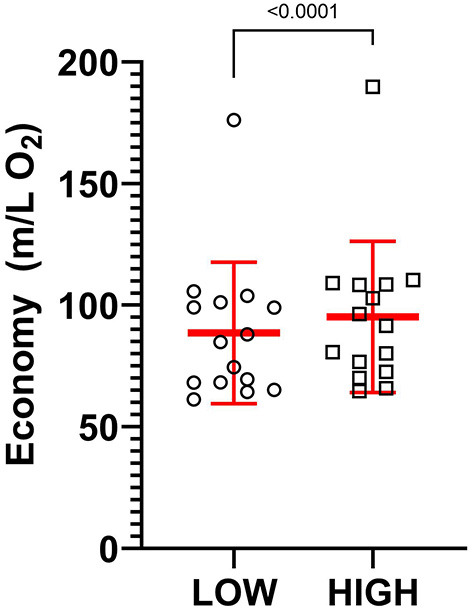
A comparison of individual and mean running economy in the LOW and HIGH support conditions during the treadmill running task. A significant increase in running economy was observed in the HIGH compared to LOW support conditions.

**Figure 6:**
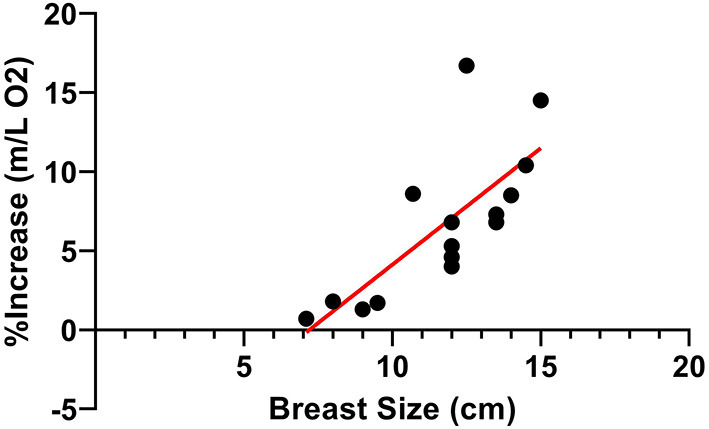
A strong, significant positive relationship was observed between participant breast size (cm) and breast support-induced increases in running economy during the treadmill running task.

Table 3 presents running economy values for each participant. The Wilcoxon test revealed greater running economy in the HIGH compared to LOW support condition (Figure 5, *p* < 0.001, Median = 6.20, *d* = 0.22). Further a significant positive correlation was observed between the increase in running economy in the HIGH compared to LOW support condition and participant breast size (Figure 6, *p* < 0.001, ρ = 0.807, ρ^2^ = 0.651).

**Table 3:** Running economy values for each participant measured as the distance traveled per liter of oxygen consumed.

**Subject**	**Breast**	**LOW**	**HIGH**	**%**
	**size (cm)**	**(m/L O_**2**_)**	**(m/L O_**2**_)**	**Increase**
S1	15.0	69.5	80.2	14.5
S2	12.0	68.3	72.6	5.3
S3	9.5	105.7	108.5	1.7
S4	12.0	61.3	64.7	4.6
S5	21.5	65.2	76.7	16.7
S6	13.5	84.9	91.5	6.8
S7	14.5	99.1	110.4	10.4
S8	10.7	88.0	96.4	8.6
S9	12.0	103.9	109.1	4.0
S10	12.0	176.1	189.8	6.8
S11	13.5	74.5	80.7	7.3
S12	14.0	99.0	108.4	8.5
S13	9.0	64.4	65.9	1.3
S14	7.1	101.2	102.9	0.7
S15	8.0	68.2	70.1	1.8
Mean	12.3	88.6	95.2	6.6
SD	3.5	29.1	31.1	4.7

*The HIGH compared to LOW support condition was associated with a 6.6% increase in running economy (p < 0.001, t = 6.30)*.

Note: ^*^ - denotes a significant difference compared to the LOW condition.

### Temporospatial Characteristics of Running

Table 4 presents temporospatial characteristics of the two treadmill running conditions including cadence, ground contact time, and step length. No differences were observed in cadence (*p* = 0.174, *t* = 1.43, *d* = 0.12), step length (*p* = 0.111, *t* = 1.70, *d* = 0.03) or ground contact time (*p* = 0.123, *t* = 0.121, *d* = 0.07).

**Table 4:** Average temporospatial characteristics in the LOW and HIGH support conditions.

**Subject**	**Breast size (cm)**	**Cadence (steps/min)**		**Step length (m)**		**Ground contact time (ms)**	
		**LOW**	**HIGH**	**LOW**	**HIGH**	**LOW**	**HIGH**
S1	15.0	163	162	0.97	0.97	246	258
S2	12.0	174	181	0.85	0.82	291	291
S3	9.5	181	182	0.95	0.94	305	303
S4	12.0	166	167	0.90	0.89	280	278
S5	21.5	150	155	1.10	1.06	303	299
S6	13.5	170	173	0.90	0.88	250	254
S7	14.5	167	166	1.24	1.25	270	268
S8	10.7	166	166	1.14	1.14	254	249
S9	12.0	175	173	1.12	1.13	235	235
S10	12.0	162	164	1.20	1.18	323	318
S11	13.5	160	158	1.20	1.22	289	284
S12	14.0	157	155	1.29	1.30	318	304
S13	9.0	168	171	1.00	0.98	297	293
S14	7.1	178	177	1.13	1.13	301	302
S15	8.0	166	168	0.75	0.74	291	291
Mean	12.3	166.9	167.9	1.05	1.04	284	282
SD	3.5	8.1	8.5	0.16	0.17	27	24

*No differences were observed in temporospatial characteristics*.

## Discussion

The findings of the current study revealed that greater breast support was associated with reduced absolute and relative oxygen consumption and greater running economy during a treadmill running task at a constant mechanical demand. These breast support-related changes in metabolic cost and efficiency occurred in the absence of changes in the temporospatial characteristics of running. Therefore, not only was the mechanical demand of the treadmill running task consistent across both breast support conditions, but the time-space geometry by which the participant elected to accommodate this mechanical demand was similar across both breast support conditions. These findings have substantial implications for recreational and competitive female athletes with regards to sport performance.

Greater breast support was associated with reduced oxygen cost and greater running economy. The current data demonstrated a 6.9% reduction in oxygen consumption when running in the high compared to low support sports bra. Therefore, participants could run faster at a similar oxygen consumption when wearing the high compared to low support sports bra. In the current study, this would equate to running at 3.1 m/s compared to 2.9 m/s, an increase of 0.2 m/s with a consistent oxygen consumption. For all participants, greater breast support was associated with reductions in metabolic cost of running (L O_2_) and improvements in running economy (m/L O_2_). One candidate mechanism by which greater breast support would improve metabolic cost of running pertains to the biomechanical patterns selected by female runners to avoid breast discomfort. Two related causes of breast discomfort include high breast velocities and forceful breast contact with the anterior trunk wall (McGhee and Steele, 2020b). The high breast velocities are induced by trunk accelerations as the GRF transient propagates through the kinetic chain following initial contact. These trunk accelerations create secondary accelerations of the proximal breast creating high breast velocities as well as high strain magnitudes and high strain rates within the breast tissue (McGhee et al., 2013). In many women, the downward movement of the breasts during the stance phase of running often results in the breasts forcefully striking the anterior trunk wall creating a secondary instance of high breast accelerations (McGhee and Steele, 2020b). These high breast velocities and accelerations are purported to underlie running-related breast discomfort (McGhee and Steele, 2020b). One strategy that could be employed to reduce trunk and breast velocities during running is to reduce stiffness (increase compliance) of the lower limb and increase attenuation of the GRF transient by the ankle, knee and hip joints (Crosslin et al., 2022).

Stiffness is a composite measure that characterizes the magnitude of load applied to a structure and the structure's response to that applied load (Butler et al., 2003; Powell et al., 2014, 2016, 2017). While many variations of stiffness exist including leg stiffness, vertical stiffness and joint stiffness, previous research has demonstrated that oxygen consumption and running economy are related to lower extremity stiffness values (McMahon and Cheng, 1990; Latash and Zatsiorsky, 1993; Kerdok et al., 2002; Butler et al., 2003; Beck et al., 2016; Moore et al., 2019). It is widely accepted that greater lower extremity stiffness is associated with improved running economy (Kerdok et al., 2002; Butler et al., 2003; Beck et al., 2016; Moore et al., 2019) due to the greater utilization of stored energy in passive, elastic tissues (Latash and Zatsiorsky, 1993). One measure of stiffness that been associated with improved mechanical and metabolic performance during a running task is lower limb and knee joint stiffness (Latash and Zatsiorsky, 1993; Butler et al., 2003; Beck et al., 2016; Powell and Williams, 2018; Moore et al., 2019). Calculated as the ratio of force applied to a structure divided by the deformation of the structure during the load attenuation of the stance phase of running, stiffness characterizes the muscular and skeletal responses of the lower limb to prevent collapse (Butler et al., 2003; Powell et al., 2014, 2016, 2017; Powell and Williams, 2018). Recent research investigating the effect of increasing levels of breast support on knee joint stiffness demonstrated that greater breast support was associated with greater knee joint stiffness values (*p* = 0.002) which were primarily mediated by reductions in the knee joint excursions (*p* < 0.001) rather than increases in knee joint moments (*p* = 0.202) (Crosslin et al., 2022). These stiffness data demonstrate that lower levels of breast support were associated with reduced lower limb stiffness (increased compliance) supporting the postulation that female runners increase lower limb compliance potentially to reduce trunk and breast velocities as well as running-related breast discomfort. These findings also suggest that increased lower limb stiffness associated with greater levels of breast support would be associated improved running performance, both mechanically and metabolically (Latash and Zatsiorsky, 1993; Butler et al., 2003; Beck et al., 2016; Moore et al., 2019).

A second mechanism by which greater levels of breast support may improve running bioenergetics including oxygen cost and running economy pertains to control of the trunk. Trunk motion has been demonstrated to influence the energetic costs of running (Schutte et al., 2015, 2018; De Brabandere et al., 2018). Using trunk-mounted tri-axial accelerometers, Schutte et al. demonstrated that the fusion of several kinematic-based variables could explain variations in energy cost of running (Schutte et al., 2018) while also detecting fatigue-induced changes in running instability (Schutte et al., 2015). Though the equations developed to assess the energy cost of running included temporospatial variables such as step and stride lengths, these algorithms also included non-linear measures of variability including sample entropy (Schutte et al., 2015, 2018). Sample entropy is a measure of signal regularity and evaluates the moment-to-moment fluctuations in a signal as opposed to the signal's central tendency (such as standard deviation or coefficient of variation). The Optimal Movement Variability Theory suggests that biological signals have inherent variability and that a range of optimal variability exists. Further, it suggests that insufficient or excessive variability is indicative of suboptimal performance of the biological system (Stergiou et al., 2006; Stergiou and Decker, 2011). Consistent with previous findings, it could be postulated that in the current study the treadmill running task resulted in greater breast motion (Scurr et al., 2010, 2011b), which altered trunk motion (Risius et al., 2017) and trunk motion variability in the LOW compared to HIGH support sports bra. Though at present, no data exists evaluating the influence of breast support on the non-linear measures of trunk motion variability used by Schutte and colleagues (Schutte et al., 2015, 2018; De Brabandere et al., 2018), emerging data supports the postulation that variability of trunk motion is greater in low- compared to high-support sports bras (Powell et al., 2022). It is possible that increases in trunk rotation variability associated with wearing a low-support sports bra would create greater instability during the running task increasing the energy cost of running. However, it should be noted that no data currently exists supporting this postulation and further investigation is warranted.

While the current findings provide novel insight into the secondary effects of breast support on oxygen consumption and running economy during a treadmill running task, several limitations should be considered. Though our *a priori* power analysis suggested that our sample size was large enough to provide sufficient statistical power, the sample size remains small which may limit generalizability to the greater population. However, the repeated measures design would increase the statistical power of these comparisons by removing a number of confounding variables. Further, our use of Cohen's *d* estimates of effects size (Cohen, 1988) provides a second measure by which the means are compared relative to the pooled variance. The small sample size may have influenced the findings of the correlation analysis and may limit the generalizability of these findings beyond the current study; however, these data clearly indicate that further investigation into the influence of sports bra support on running performance and bioenergetics is warranted. A second limitation pertains to the participant's breast sizes. Specifically, no statistical adjustments were made to account for breast size; however, the correlation analyses highlighted the positive relationship between breast size and breast support-related changes in running performance. Moreover, the strong correlations and large coefficients of determination indicate the meaningful effect of breast support on these measures of oxygen consumption and running economy. Further, our participant cohort was fairly homogenous with smaller breast sizes than those previously studied. A vast majority of research investigating the effects of breast support on running biomechanics and energetics has focused on large breasted women with cup sizes of D or greater. However, the participant cohort in our study had breast sizes between B and DD cup, a potentially more representative sample of competitive and recreational athletes. Though the athletes participating in this study had smaller breast sizes than previous research studies, our data revealed that even smaller breasted females experience improved performance with greater breast support. While, the methods used to quantify breast size in the current study are commonly used in American retail (McGhee and Steele, 2010b) establishments, it has been suggested to poorly measure the size and characteristics of the female breast (Coltman et al., 2017). Though better techniques that would more adequately measure the volume of the breast and better characterize the mechanical characteristics of the breast are in development, the measurement methods used in this study represents one current standard in determining breast size (McGhee and Steele, 2010b). A final limitation of the current investigation was the limited objective data regarding the support provided by each of the sports bras used. Though breast motion can be tracked using a variety of measurement techniques, no systematic study of the support offered by these sports bras has been conducted.

## Conclusions

The findings of the current study demonstrate that greater breast support is associated with improved absolute and relative oxygen consumption as well as running economy. Further, the benefits of increased breast support on oxygen consumption and running economy are influenced by breast size with larger breasted athletes seemingly experiencing greater improvements in running performance than smaller breasted women. These improvements in running performance with increasing breast support show that sports bras should be considered a key component in female athletes' sports equipment and care should be taken to select the proper level of breast support.

## Data Availability Statement

The original contributions presented in the study are included in the article/supplementary material, further inquiries can be directed to the corresponding author/s.

## Ethics Statement

The studies involving human participants were reviewed and approved by University of Memphis Institutional Review Board (PRO-FY2020-431). The patients/participants provided their written informed consent to participate in this study. Written informed consent was obtained from the individual(s) for the publication of any potentially identifiable images or data included in this article.

## Author Contributions

Both authors were involved in study conception and design. HF collected data. HF and DP performed data analysis. HF and DP prepared the initial draft of the manuscript. Both authors revised, edited, and approved the final manuscript.

## Funding

This project received financial support from the American Athletic Conference Research Consortium.

## Conflict of Interest

The authors declare that the research was conducted in the absence of any commercial or financial relationships that could be construed as a potential conflict of interest.

## Publisher's Note

All claims expressed in this article are solely those of the authors and do not necessarily represent those of their affiliated organizations, or those of the publisher, the editors and the reviewers. Any product that may be evaluated in this article, or claim that may be made by its manufacturer, is not guaranteed or endorsed by the publisher.
